# Maryland Medical Journal

**Published:** 1877-06

**Authors:** 


We are glad to see among our exchanges a copy of the *Maryland Medical Journal*, edited by Drs. Manning and Ashley, of Baltimore. It is gotten up in nice form, and is replete with valuable information. May success attend it.

